# Analysis of Genetic Determinants Encoding Resistance to Heavy Metals and Disinfectants in *Listeria monocytogenes*

**DOI:** 10.3390/foods13233936

**Published:** 2024-12-06

**Authors:** Elżbieta Maćkiw, Joanna Kowalska, Dorota Korsak, Jacek Postupolski

**Affiliations:** Department of Food Safety, National Institute of Public Health NIH-National Research Institute, 24 Chocimska str, 00-791 Warsaw, Poland; jkowalska@pzh.gov.pl (J.K.); dkorsak@pzh.gov.pl (D.K.); jpostupolski@pzh.gov.pl (J.P.)

**Keywords:** *Listeria monocytogenes*, heavy metals, disinfectants, cadmium, arsenic, benzalkonium chloride, resistance determinants

## Abstract

*Listeria monocytogenes* is an important foodborne pathogen causing listeriosis. *L. monocytogenes*, existing in the natural environment, can also contaminate food products, which poses a serious threat to human health and life, especially for high-risk groups: pregnant women, newborn babies, and the elderly. Environmental adaptation of *L. monocytogenes* refers to the various strategies and mechanisms used by this bacterium to survive and thrive in diverse and often hostile environments that include, among others, toxic heavy metals and disinfectants. The aim of this study was to analyze WGS (whole-genome sequencing) data of 45 *L. monocytogenes* strains isolated from food to compare the prevalence and types of genetic determinants encoding resistance to toxic metals, such as arsenic and cadmium, as well as quaternary ammonium compounds, like benzalkonium chloride. In *L. monocytogenes* strains, resistance genes were detected for disinfectants, such as benzalkonium chloride (4.4%), as well as for toxic heavy metals, like cadmium (28.9%) and arsenic (24.4%). The *bcrABC* cassette was found together with the *cadA2C2* genes in two strains: 3855-D (IIc, ST9, CC9) and 4315 (IVb, ST6, CC6). The arsenic cassette, encoded by the genes *arsR1D2R2A2B1B2*, was co-selected with the *cadA4C4* genes. The arsenic cassette was prevalent in nine strains of clonal complex CC2 (82%), one strain of CC3 (9%), and one strain of CC11 (9%). In contrast, the benzalkonium chloride cassette was detected in one strain of CC6 and one strain of CC9. The results of the present study demonstrate the need for further research into the characteristics of *L. monocytogenes* isolated from other sources in order to understand their spread throughout the food chain.

## 1. Introduction

*Listeria monocytogenes* is the causative agent of listeriosis in humans and animals, representing one of the major foodborne pathogens, with high rates of mortality of 20–30% [1]. The groups at highest risk of listeriosis infection include pregnant women, newborn babies, the elderly, and people with weakened immune systems resulting from illness or medication. Listeriosis outcomes include septicemia, meningitis, stillbirth, and miscarriages [2,3,4]. In 2022, a total of 2738 confirmed cases of listeriosis were reported, with a case fatality rate of 18.1%. Infections were most frequently reported in the age group >64 years, accounting for 70.9% of all reported cases. The proportion of listeriosis cases has been steadily increasing over the past few years [5]. Strains of certain serotypes (1/2a, 1/2b, and 4b) are responsible for the majority (over 95%) of clinical cases in humans. Serotype 4b strains are responsible for about 50% of human listeriosis cases.

*L. monocytogenes* is a bacterium widely distributed in the natural environment; it can be found in water, soil, rotting plants, and sewage. Due to its resistance to environmental conditions, it can also contaminate food-processing areas and foodstuffs.

The most commonly affected food products are ready-to-eat (RTE) items, including smoked fish, soft cheeses, deli meats, and salads. It is estimated that nearly 90% of human cases of listeriosis worldwide occur after consuming contaminated food [6,7].

The environmental adaptation of *L. monocytogenes* is indeed a complex and multifaceted process. This bacterium has evolved multiple mechanisms to adapt to a wide range of stressors, including temperature extremes, pH fluctuations, the presence of antimicrobial agents, disinfectants, and heavy metals.

Disinfectants as quaternary ammonium compounds (QACs), such as benzalkonium chloride (BC), benzethonium chloride (BZC), and cetyltrimethylammonium bromide (CTAB), are a class of antimicrobial agents widely used in the food industry [8]. Their primary function is to control microbial contamination, ensuring food safety and extending shelf life [9]. BC exhibits good antibacterial activity against many important foodborne pathogens [10]. However, some studies have indicated that the use of certain disinfectants has imposed a selective pressure and contributed to the emergence of disinfectant-resistant microorganisms in food environments. When resistance genes are located on mobile genetic elements (MGEs), such as plasmids or transposons, they can potentially be transferred horizontally between bacteria, leading to the dissemination of resistance phenotypes. Recent studies show that *L. monocytogenes* is resistant to various disinfectants, including organic acids and quaternary ammonium compounds, which poses a serious challenge to the food industry [8]. Several different QAC resistance determinants associated with MGEs leading to increased BC tolerance in *L. monocytogenes strains* have been discovered: (i) the *bcrABC* cassette located within a putative composite transposon, which is present in many plasmids, e.g., pLM80 [11]; (ii) *qacH* encoded within transposon Tn*6188* [12]; (iii) *emrE* in a genomic island (LGI-1) [13]; and (iv) the *emrC* gene present in plasmid pLMST6 [14].

As previously mentioned, one key aspect of *L. monocytogenes’* environmental adaptation is its complex ability to resist heavy metals. Metals, such as arsenic and cadmium, are naturally occurring chemical compounds. They can be present at various levels in the environment, e.g., the soil, water, and the atmosphere. Metals can also occur as residues in food because of their presence in the environment as a result of human activities, such as farming, industry, or car exhausts, or from contamination during food processing and storage [15]. As documented by Nies [16], *L. monocytogenes* has the ability to survive in environments with high heavy metal content, which may contribute to its survival in polluted industrial areas.

The presence of heavy metal ions in the environment leads bacteria to develop protective mechanisms that enable them to survive in the presence of toxic concentrations. Resistance to heavy metals, especially arsenic and cadmium, has long been recognized as a major adaptation of *L. monocytogenes* [17,18].

Cadmium resistance determinants are widely distributed and commonly associated with *L. monocytogenes* strains isolated from different sources [19]. To date, seven different determinants of cadmium resistance (*cadAC* efflux systems) have been identified in *Listeria* spp. The CadA protein, a P-type ATPase efflux pump, actively transports cadmium ions out of bacterial cells, thereby lowering the intracellular cadmium concentration. Meanwhile, CadC serves as a regulatory protein that governs the expression of the *cadA* gene. By sensing toxic compounds in the environment, CadC activates the transcription of *cadA*, increasing the production of the CadA efflux pump as necessary [20]. The *cadA1* gene is located within transposon Tn*5422* (Tn*3* family) and commonly inserted within plasmids [21]. A second variant of the *cadA* gene, *cadA2*, is often found on plasmids and typically associated with the *bcrABC* cassette [11,22,23]. The third cadmium resistance gene, *cadA3,* was found in chromosome *L. monocytogenes* EGD-e located within the integrative and conjugative element ICELm1 (Tn*916*-like) [24]. Regarding the *cadA4* and *cadA5* genes, so far, only one has been identified on the large chromosomally located *Listeria* Genomic Island 2 (LGI2) and LGI2-1 island, respectively [25,26]. The first three variants confer a high level of resistance to cadmium (MIC > 140 μg/mL), whereas *cadA4* is responsible for relatively lower resistance levels (MIC < 70 μg/mL) [6]. The new variant cassette *cadA6* was detected in *Listeria* species, including strains of *L. monocytogenes,* isolated from various countries and sources. Four new spliced or unspliced transposons in plasmids and chromosomes maintain this cassette [27]. Recently, a novel cadmium resistance gene, *cadA7*, was found in a transposon Tn*916* variant inserted in the chromosome *L. monocytogenes* strain [28]. The *CadA7* gene has been also detected on a novel LGI2 variant, LGI2-3, in two *L. welshimeri* strains [29].

Arsenic resistance in *Listeria* spp. in most cases is chromosomally encoded, either within aTn*554*-like transposon (*arsCBADR*) or genomic islands LGI2 and LGI2-1 (*arsR1D2R2A2B1B2* and upstream *arsA1D1* cassette) [24,25]. However, the occurrence of arsenic resistance genes within *Listeria* spp. plasmids has also been observed [24,30,31].

The aim of this study was to analyze WGS data of 45 *L. monocytogenes* strains isolated from food to identify the genome characteristics and compare the prevalence and types of genetic determinants encoding resistance to toxic metals, such as arsenic and cadmium, as well as quaternary ammonium compounds, like benzalkonium chloride.

## 2. Materials and Methods

### 2.1. L. monocytogenes Strains

*Listeria monocytogenes* strains were isolated from retail food samples in the frame of the Official Control and Monitoring Program by Sanitary and Epidemiological Stations, in accordance with the PN-EN ISO 11290-1 [32] or PN-EN ISO 11290-2 [33] method accredited by the Polish Centre of Accreditation. *L. monocytogenes* isolates were sent to the National Reference Laboratory for *L. monocytogenes* for confirmation tests. The *L. monocytogenes* isolates were stored at −70 °C until further analysis in Brain Heart Infusion medium with 20% sterile glycerol. One strain from the same food matrix, isolated the same year, was selected for whole genome sequencing (WGS) and genomic analysis. In total, 45 isolates from different food products were included.

### 2.2. DNA Isolation

DNA was isolated using of the E.Z.N.A.^®^ Bacterial DNA Kit (Omega Bio-Tek, Norcross, GA, USA) according to the recommendations of the manufacturer. The DNA concentration was determined by NanoDrop OneC (Invitrogen, Thermo Fisher Scientific, Waltham, MA, USA).

### 2.3. Whole Genome Sequencing (WGS)

Sequencing was conducted by Genomed (Warsaw, Poland) using MiSeq paired-end (PE) technology, 2 × 300 nt, with the MiSeq Reagent Kit v3, 600-cycle (Illumina), as per the manufacturer’s protocol. The genomic DNA concentration was measured prior to the library preparation process using Pico-Green reagent (Life Technologies, Thermo Fisher Scientific) with a Tecan Infinite device. Genomic DNA was fragmented using sonication with the Covaris E210, following the recommended parameters for preparing libraries for Illumina sequencing technology. Libraries were then prepared with the NEBNext Ultra II DNA Library Prep Kit for Illumina (New England Biolabs, Ipswich, MA, USA) following the manufacturer’s instructions.

### 2.4. Genome Assembly and WGS Quality Control

Readings were filtered with Cutadapt version 3.0. Quality control of the sequencing results was performed using FastQC software Galaxy Version 0.72+galaxy1. *Denovo* assembly was performed with Spades version 3.14.5. For sequence data, an average depth coverage of over 50× was required. The genome size, ranging between 2.8 and 3.1 Mb and consistent with *L. monocytogenes* parameters, was used as a criterion for assembly quality. Sequences of isolates were submitted to the NCBI GenBank (accession Bio-project numbers: PRJNA1187899).

### 2.5. MLST, cgMLST

The MLST (Multi-Locus Sequence Typing) and cgMLST (core genome Multi-Locus Sequence Typing) method for *L. monocytogenes* was performed using an assembly-based sequence in Ridom SeqSphere+. The identity percentage was set at 90%. The analysis was performed following the Ruppitsch scheme [34] with the seed genome EGD-e (NC_003210.1, 17-DEC-2014).

### 2.6. SNP Analysis

Single Nucleotide Polymorphism (SNP) analysis was conducted using the CSI Phylogeny 1.4 tool from the Center for Genomic Epidemiology (CGE), accessed at www.genomicepidemiology.org accessed on 15 April 2024. Phylogenetic trees were generated based on our datasets using our first genome as the reference. The resulting Newick files were visualized with iTol https://itol.embl.de/ accessed on 15 April 2024.

### 2.7. In Silico Detection of Heavy Metals and Disinfectant Resistance Genes

The presence of arsenic, cadmium, and benzalkonium chloride resistance genes (*casette arsD1A1R1D2R2A2B1B2*, *cassette arsCBADR*, *cassette bcrABC*, *qacH*, *emrE*, *cadA1cadC1*, *cadAcadA2*, *cad3cadA3*, and *cadA4cadC4*) was detected using Ridom SeqSphere+ softwareVersion 10.0.4. The analysis was conducted with default settings, requiring a reference sequence identity of at least 90% and a base sequence identity of 99%. The presence of plasmids was searched for with the PlasmidFinder v2.0 tool at CGE DTU servers [35]. The sequences of the *L. monocytogenes* strain were annotated using Prokka Galaxy Version 1.14.6+galaxy1 software on the usegalaxy server. BLAST (https://blast.ncbi.nlm.nih.gov/, accessed on 30 July 2024) was used to verify the presence of MGEs using GenBank sequences HG329628, AADR01000010, LT732640, L28104, FR33648, and CM001159.

## 3. Results and Discussion

In our study, *L. monocytogenes* isolates were differentiated into 19 sequence type STs grouped into 15 clonal complexes (CCs) based on the MLST analysis: ST2 (CC2; 15.6%), ST145 (CC2; 15.6%), ST6 (CC6; 11.1%), ST9 (CC9; 6.7%), ST121 (CC121; 6.7%), ST37 (CC37; 6.7%), ST155 (CC155; 6.7%), 2.2% ST8 (CC8; 4,4%), ST10 (CC101; 2,2%), ST16 (CC8; 2,2%), ST20 (CC20; 2.2%), ST91 (CC14; 2.2%), ST101 (CC101; 2.2%), ST193 (CC193; 2.2%), ST394 (CC415; 2.2%), ST412 (CC412; 2.2%), ST3 (CC3; 2.2%), ST451 (CC11; 2.2%), and ST124 (4.4%). Some of these CCs are the most common clones in Europe, such as CC2, CC121, CC9, CC8, CC6, and CC155 [1]. The majority of isolates (n = 25) belonged to lineage II, and 20 isolates belonged to lineage I. This is in agreement with the results of Brown et al. [4].) However, in that study, the percentage of isolates belonging to lineage II was significantly higher (93%). In our study, the dominant serogroups were IIa (n = 22; 48.9%) and IVb (n = 18; 40%), with the remaining serogroups being IIc (n = 3; 6.67%) and IIb (n = 2; 4.4%). Among lineage II isolates, strains belonging to CC2 (n = 14; 31.1%) were clearly predominant (Table 1).

**Figure 1:**
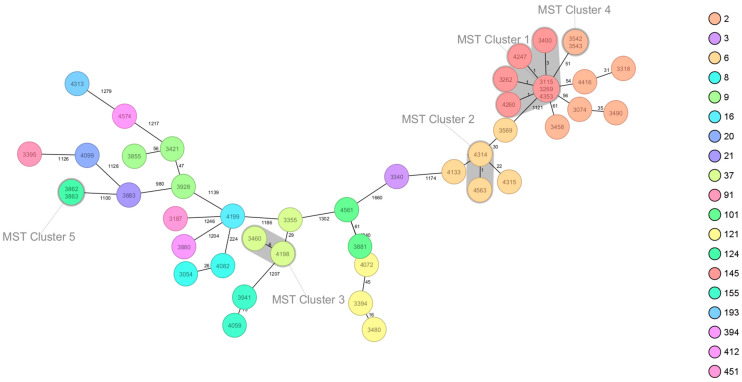
The 45 strains of *L. monocytogenes* isolated from food in Poland. Minimum spanning tree of cgMLST allelic profiles. Each circle represents a particular cgMLST type. The distance between the circles represents the genetic divergence. The divergence is given in the number of allelic differences and is indicated on the branch. Colors represent Multi-Locus Sequence Typing (MLST) clonal complexes. The clusters are marked with a loop. The circle label gives the strain identifier and the strain MLST sequence type. Trees of *L. monocytogenes* were generated using Ridom Seqsphere+.

The cgMLST analysis performed revealed the presence of five clusters. MST cluster 1 (seven isolates—3115, 3269, 4353, 4247, 3262, 4260, 3400; belonging to serogroup IVb, CC2 and ST145), cluster 2 (two isolates—4314, 4563; belonging to serogroup IVb, CC6 and ST6), cluster 3 (two isolates—4198, 3460; belonging to serogroup IIa, CC37 and ST37), cluster 4 (two isolates—3542, 3543; belonging to serogroup IVb, CC2 and ST2), and cluster 5 (two isolates—3862, 3863; belonging to serogroup IIa, ST124) (Figure 1).

**Figure 2:**
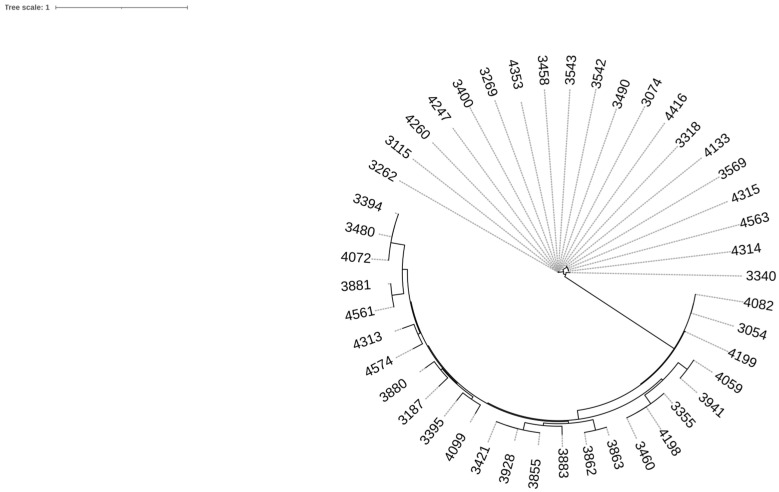
SNP phylogeny of *L. monocytogenes* using the CSI Phylogeny 1.4 tool provided by the Center for Genomic Epidemiology, accessed at www.genomicepidemiology.org accessed on 15 April 2024. We generated phylogenetic trees based on our datasets and used our first genome as a reference. To visualize the Newick files that were generated, we utilized iTol (https://itol.embl.de/) accessed on 15 April 2024.

The visualized SNP phylogeny of the *L. monocytogenes* results is shown in Figure 2. Interestingly, the pairwise SNP distances between some isolates were small. The genetic differences within some genomes ranged from two to nine pairwise SNP differences, and they are as follows: two SNPs between isolates: 4247 and 4260, 4247 and 4353, and 4353 and 4260; four SNPs: 3542 and 3543; six SNPs: 3863–3862; eight SNPs: 3460–4198; nine SNPs: 3115, 4247, 4260, 4353; 4247–3115; 4260–3115; 4353–3115. This range indicates that the samples are very closely related, suggesting a common origin or recent separation from a common ancestor. In the context of cluster analysis, such small differences in SNPs can indicate that samples belong to the same epidemiological cluster, which is particularly important for tracking disease outbreaks and establishing links between cases. As products have been sampled over several years and strains have been consistently isolated, this suggests that *Listeria* strains have been continuously present in the food production chain. This may indicate that there are strains that persist in production facilities.

In our study, specific resistance genes were detected in phylogenetically distant lineages I and II. In addition, the presence of resistance genes to arsenic, cadmium, and benzalkonium chloride was associated with specific serotypes and CCs. The reference list used in building the scheme in Ridom SeqSphere+ is presented in Table 1. In the present study, the occurrence of cadmium resistance genes was most prevalent in strains of serogroups IVb and CC2 (n = 9); furthermore, resistance genes were found in the strain of serogroup IVb and CC6 (n = 1), the strain of serogroup IIb and CC3 (n = 1), the strain of serogroup IIa and CC11 (n = 1), and the strain of serogroup IIc and CC9 (n = 1). Similarly, as regards the presence of genes encoding arsenic resistance, strains belonging to serogroup IVb and CC2 (n = 9) are most frequently affected. These genes were also detected in strains belonging to serotypes IIb and CC3 (n = 1) and serotype IIc and CC9 (n = 1). In contrast, benzalkonium chloride resistance genes have only been detected in one strain of serogroup IVb (CC6) and one strain of serogroup IIc (CC9). The results of our study are consistent with the results of previous studies [6,36]. In the study by Gelbicova et al., cadmium and arsenic resistance genes were most frequently identified in *L. monocytogenes* lineage I strains (27.7% and 16.8%, respectively). Moreover, in these studies, cadmium and arsenic resistance genes were also detected in lineage II strains (28.5% and 14.6%, respectively).

Resistance genes for disinfectants, such as benzalkonium chloride (4.4%), and heavy metals, such as cadmium (28.9%) and arsenic (24.4%), were detected in *L. monocytogenes* isolates. These results are similar to those obtained in a study of *L. monocytogenes* strains isolated from food by Gelbicowa et al. [36], where resistance genes were detected for cadmium (36.8%), arsenic (23.6%), and benzalkonium chloride (9.4%). The benzalkonium chloride resistance cassette, *bcrABC*, was identified in two strains, 3855 (IIc, ST9, CC9) and 4315 (IVb, ST6, CC6), and it was found together with the *cadA2C2* genes. As described by Elhanafi et al. [11], *bcrABC* was first isolated from strains involved in the 1998–1999 foodborne outbreaks. Dutta et al. [22] showed that bcrABC sequences are highly conserved among *L. monocytogenes* strains of different serotypes, with variability mainly in the flanking regions. Most BCr isolates showed a pLM80-like organization of the bcrABC region, while others contained it chromosomally. The role of the *bcrABC* cassette in BC resistance exhibited by *L. monocytogenes* isolated from food processing environments has been reported in numerous studies [11,19,37,38,39]. Our study confirms that *L. monocytogenes* isolates resistant to BC and carrying the *bcrABC* cassette are also resistant to cadmium. This cross-resistance, likely mediated by the co-occurrence of cadA2C2 genes, suggests a widespread distribution of *bcrABC* in *L. monocytogenes*, independent of serotype or source, and highlights potential mechanisms for its horizontal spread. Our results support the hypothesis of horizontal gene transfer of *bcrABC,* as proposed by Dutta et al. [22], while emphasizing its association with other resistance genes, such as *cadA2.* The strains carried the *cadA2* gene either alone or together with the *cadA1* gene [22]. The arsenic cassette arsR1D2R2A2B1B2, found together with *cadA4C4* genes, was detected in nine isolates of the serotype IVb (3262, 3269, 4247, 4260, 3115, 4353, 3074, 3458, 4416), one of IIa (3187), and one of IIb (3340). This simultaneous resistance to arsenic and cadmium is mediated by a 35-kb chromosomal region (LGI-2). LGI-2 also contains genes associated with DNA integration, conjugation, and pathogenicity, making it a critical region for both resistance and virulence [26]. LGI-2 was originally identified in serogroup 4b CC2 strain Scott A [25,26,39]. As shown in recent studies, arsenic and cadmium resistance encoded by genes carried on LGI-2 is strongly associated with its presence in the *L. monocytogenes* serogroup 4b strains, particularly in the hypervirulent clones CC1, CC2, and CC4. This finding is corroborated by the work of Lee et al. [25], who also identified LGI-2 in these strains as a significant factor contributing to both resistance and virulence. In our study, out of nine serogroup 4b strains, seven belong to clones CC2 and one to CC4.

The emergence of *L. monocytogenes* strains carrying both disinfectant and heavy metal resistance genes, particularly in food processing environments, is likely to be driven by the selective pressure of frequent exposure to disinfectants and metals. Studies suggest that these environments act as reservoirs for resistant strains, promoting their persistence and potential to cause outbreaks [22].

## Figures and Tables

**Table 1:** *L. monocytogenes* isolate characterization and genetic determinants encoding resistance to toxic metals (cadmium and arsenic) and quaternary ammonium compounds (benzalkonium chloride).

Strain ID	Lineage	Serogrup	Genes									Localization	Sequence Type	Clonal Complex	MST Cluster	Year of Isolation	Food Type
			*bcrABC*	*emrE*	*qacH*	*cadA1cadC1*	*cadA2cadC2*	*cadA3cadC3*	*cadA4cadC4*	*arsD1A1R1D2R2A2B1B2*	*arsCBADR*						
3074	I	IVb	-	-	-	-	-	-	+	+	-	chromosome—Genomic Island LGI2	ST2	CC2		2016	raw cabbage salad
3115			-	-	-	-	-	-	+	+	-	chromosome—Genomic Island LGI2	ST145	CC2	1	2018	vegetable salad
3262			-	-	-	-	-	-	+	+	-	chromosome—Genomic Island LGI2	ST145	CC2	1	2017	dumplings with cottage cheese
3269			-	-	-	-	-	-	+	+	-	chromosome—Genomic Island LGI2	ST145	CC2	1	2017	pastries
3400			-	-	-	-	-	-	-	-	-		ST145	CC2	1	2017	strawberry sorbet
3458			-	-	-	-	-	-	+	+	-	chromosome—Genomic Island LGI2	ST2	CC2		2017	dumplings with potato and cheese
3490			-	-	-	-	-	-	-	-	-		ST2	CC2		2018	milk ice cream
3542			-	-	-	-	-	-	-	-	-		ST2	CC2	4	2018	fruity cake
3543			-	-	-	-	-	-	-	-	-		ST2	CC2	4	2018	fruity cake
3569			-	-	-	-	-	-	-	-	-		ST6	CC6		2017	frozen corn
4133			-	-	-	-	-	-	-	-	-		ST6	CC6		2018	tartare
4247			-	-	-	-	-	-	+	+	-	chromosome—Genomic Island LGI2	ST145	CC2	1	2018	smoked salmon
4260			-	-	-	-	-	-	+	+	-	chromosome—Genomic Island LGI2	ST145	CC2	1	2018	pastries
4314			-	-	-	-	-	-	-	-	-		ST6	CC6	2	2018	smoked mackerel
4315			+	-	-	-	+	-	-	-	-	plasmid pLM80-like	ST6	CC6		2018	smoked salmon
4353			-	-	-	-	-	-	+	+	-	chromosome—Genomic Island LGI2	ST145	CC2	1	2019	dumplings with meat
4416			-	-	-	-	-	-	+	+	-	chromosome—Genomic Island LGI2	ST2	CC2		2019	pastries
4563			-	-	-	-	-	-	-	-	-		ST6	CC6	2	2019	trout fillet
3318		IIb	-	-	-	-	-	-	-	-	-		ST2	CC2		2017	cake
3340			-	-	-	-	-	-	+	+	-	chromosome—Genomic Island LGI2	ST3	CC3		2017	cake
3054	II	IIa	-	-	-	-	-	-	-	-	-		ST8	CC8		2016	broccoli pasta salad
3187			-	-	-	-	-	-	+	+	-	chromosome—Genomic Island LGI2	ST451	CC11		2017	tartar label
3355			-	-	-	-	-	-	-	-	-		ST37	CC37		2017	smoked mackerel paste
3394			-	-	-	-	-	-	-	-	-		ST121	CC121		2017	smoked salmon
3395			-	-	-	-	-	-	-	-	-		ST91	CC14		2017	white turnip salad
3460			-	-	-	-	-	-	-	-	-		ST37	CC37	3	2017	dumplings with meat
3480			-	-	-	-	-	-	-	-	-		ST121	CC121		2017	smoked salmon
3862			-	-	-	-	-	-	-	-	-		ST124	-	5	2018	vegetable mix
3863			-	-	-	-	-	-	-	-	-		ST124	-	5	2018	vegetable mix
3880			-	-	-	-	-	-	-	-	-		ST394	CC415		2018	vegetables for frying
3881			-	-	-	-	-	-	-	-	-		ST101	CC101		2018	frozen corn
3883			-	-	-	-	-	-	-	-	-		ST155	CC155		2018	frozen stir fry vegetable
3941			-	-	-	-	-	-	-	-	-		ST155	CC155		2018	vegetable salad
4059			-	-	-	-	-	-	-	-	-		ST155	CC155		2018	ice cream
4072			-	-	-	-	-	-	-	-	-		ST121	CC121		2018	smoked salmon
4082			-	-	-	-	-	-	-	-	-		ST8	CC8		2018	pork sausages
4099			-	-	-	-	-	-	-	-	-		ST20	CC20		2018	smoked salmon
4198			-	-	-	-	-	-	-	-	-		ST37	CC37	3	2018	chicken fillet
4199			-	-	-	-	-	-	-	-	-		ST16	CC8		2018	cabbage salad
4313			-	-	-	-	-	-	-	-	-		ST193	CC193		2018	smoked mackerel
4561			-	-	-	-	-	-	-	-	-		ST10	CC101		2019	trout fillet
4574			-	-	-	-	-	-	-	-	-		ST412	CC412		2019	frozen red pepper
3421		IIc	-	-	-	-	-	-	-	-	-		ST9	CC9		2017	raw sausage
3855			+	-	-	-	+	-	-	-	-	plasmid pLM80-like	ST9	CC9		2018	chicken sausages
3928			-	-	-	-	-	-	-	-	-	plasmid pLM80-like	ST9	CC9		2018	ice cream

CC (-) not designated; (+) presence of the gene; (-) absence of the genes.

## Data Availability

The original contributions presented in the study are included in the article, further inquiries can be directed to the corresponding author.
